# Factors Associated with the Dual Use of Electronic Cigarettes and Cigarettes among Thai Undergraduate Students Who Smoked Cigarettes

**DOI:** 10.3390/children8121197

**Published:** 2021-12-17

**Authors:** Phantara Chulasai, Purida Vientong, Surarong Chinwong, John J. Hall, Dujrudee Chinwong

**Affiliations:** 1 PhD’s Degree Program in Pharmacy, Faculty of Pharmacy, Chiang Mai University, Chiang Mai 50200, Thailand; panthara.chula@gmail.com; 2 Department of Social Pharmacy, Faculty of Pharmacy, Payap University, Chiang Mai 50000, Thailand; 3 Department of Pharmaceutical Care, Faculty of Pharmacy, Chiang Mai University, Chiang Mai 50200, Thailand; purida.v@cmu.ac.th (P.V.); surarong@gmail.com (S.C.); 4 Center of Excellence for Innovation in Analytical Science and Technology for Biodiversity-Based Economic and Society (I-ANALY-S-T_B.BES-CMU), Chiang Mai University, Chiang Mai 50200, Thailand; 5 School of Population Health, University of New South Wales, Sydney 2052, Australia; john.hall@unsw.edu.au

**Keywords:** electronic cigarette, e-cigarette, e-cigarette use, cigarette, cigarette smoker, dual use, dual user, undergraduate student, undergraduate smokers

## Abstract

This study proposed to identify factors associated with the dual use of electronic cigarettes (e-cigarettes) and cigarettes among undergraduate students who smoked cigarettes. This cross-sectional study employed a self-administered, anonymous online questionnaires to collect information from undergraduate smokers in northern Thailand. Of the 494 participants, 82.8% were dual users of e-cigarettes and cigarettes. The two main reasons for using e-cigarettes were an absence of cigarette smoke odor (76.8%) and availability of flavors (70.7%). Undergraduate smokers who initiated smoking at ≥18 years old were more likely to be dual users than those who initiated smoking at younger age (adjusted odds ratio [aOR]: 2.79, 95% confidence intervals [CI]: 1.32–5.89, *p* = 0.007). Undergraduate smokers who smoked ≥11 cigarettes daily were more likely to be dual users than those who smoked less (aOR: 2.64, 95% CI: 1.52–4.61, *p* = 0.001). Conversely, undergraduate smokers who had attempted to quit during the past year were less likely to be dual users (aOR: 0.26, 95% CI: 0.12–0.56, *p* = 0.001). In conclusion, dual use of e-cigarettes and cigarettes among undergraduate smokers was associated with older age at cigarette smoking initiation, a higher number of cigarettes smoked daily, and having no past year’s cigarette quit attempts.

## 1. Introduction

An electronic cigarette (e-cigarette) is a device with a heating element that warms a liquid solution that typically consists of nicotine, flavorings, and other substances in an aerosol that is inhaled by the user [1,2]. The use of e-cigarettes among the younger populations has risen rapidly since the introduction of this product to China in 2004 [3], the US in 2006 [3], and Thailand in 2010 [4]. Several national surveys have found that the percentage of e-cigarette use, as well as the dual use of e-cigarettes and cigarettes, has increased among young people [1]. In the US, young adults aged 18–24 years had the highest increase in the prevalence of current e-cigarette use, from 9.2% in 2016 to 15.0% in 2018 [5]. In 2020, Thai university students reported e-cigarette use of 18.1% [6]. France university students reported 36.4% having tried e-cigarettes and 3.6% currently using e-cigarettes. Moreover, 5.0% of those who smoked cigarettes used e-cigarettes [7]. Australian young adults reported e-cigarette use of 9.0%. In addition, 19.0% of those who smoked cigarettes used e-cigarettes [8].

As e-cigarettes gain increasingly widespread acceptance, particularly among younger populations, it has become a challenging public health concern. While completely replacing conventional cigarettes with e-cigarettes lessens users’ exposure to toxicants and carcinogens found in cigarettes [9], e-cigarette may considerably raise the risk of cardiovascular and noncancer lung disease [10]. Furthermore, because e-cigarettes have been available for just over a decade, the prolonged health implications of e-cigarette use or dual use have not yet been completely established. Moreover, limited evidence exists to establish the potential of e-cigarettes as tobacco use cessation aids at the population level [1,3,11].

E-cigarette use is a common correlate of cigarette smoking [12,13]. Related studies have shown that while adults began using e-cigarettes to help smoking cessation [13,14,15,16], the use of e-cigarettes by young adults differs. They do not use e-cigarettes primarily to reduce or quit smoking. They are more often used concurrently with cigarettes due to self-curiosity and enjoyment [7,8,13,17,18]. Furthermore, sufficient and strong evidence exists to indicate that e-cigarette use is related with a greater risk of subsequent onset of cigarette smoking and current cigarette smoking among young adults [19,20,21,22,23].

While recent studies in Thailand have focused on e-cigarette use only, a significant gap remains in understanding the dual use among Thai undergraduate students. Therefore, this study purposed to identify factors associated with the dual use of e-cigarettes and cigarettes among undergraduate students who smoked cigarettes in Thailand.

## 2. Materials and Methods

### 2.1. Study Design and Participants

This cross-sectional study was performed on undergraduate students who were cigarette smokers from four universities in Chiang Mai Province, northern Thailand. Recruitment occurred between December 2018 and February 2019 using convenience sampling method including the snowball sampling technique; existing participants provided referrals to their friends to join this study. Eligibility to participate in this study included undergraduate students who were cigarette smokers, aged 18 years or older, could access the online questionnaires using the Google Forms platform, could communicate in Thai, and were interested to participate in this research. Participants in this study received no compensation.

### 2.2. Sample Size Calculation

The study sample size was determined using Yamane’s formula for estimating sample size for a limited population [24]. When this study was conducted at four universities in the academic year 2018, the number of undergraduate students enrolled was 68,105 [25]. The smoking rate was 20.40% [26], implying that 13,893 were smokers. As a result, with a margin of error of 0.05, the sample size for this study consisted of 389 undergraduate students who were cigarette smokers.

### 2.3. Questionnaire Development and Data Collection

First, the paper-based questionnaire was created (File S1). The questionnaire was developed based on a review of relevant publications in the related literature. A preliminary version of the questionnaire was reviewed by three experts in the area of smoking behaviors to validate its content and was piloted among 31 participants who were not engaged in the study to test language suitability. The questionnaire was revised according to the feedback of experts and participants. The online questionnaire was then revised and verified before being used in this study.

Self-administered online questionnaires, developed using the Google Forms platform, were used to collect data from anonymous participants. A hyperlink and QR code for the online questionnaire were distributed using social networks. After accessing the online questionnaire, participants obtained a study description and contact information of the researcher. Participants were instructed that they could independently disengage at any time without explanation. Only participants meeting the criteria and confirming their acceptance to participate in this study by affirming online informed consent were able to access the full questionnaire. The questionnaire was set to answer each item by proceeding to the next one until the end of the survey (approximately 15 min). Only a complete questionnaire could be sent back to the research team in this regard. Participants completing the questionnaire were encouraged to invite their friends to participate in this study.

### 2.4. Measures

This study comprised the subsets of measures described below.

#### 2.4.1. Cigarette Smoking and E-Cigarette Use Status

Participants were defined as cigarette or noncigarette smokers based on their responses to the question, “How would you describe your cigarette smoking habits?”. When they answered “smoking”, they were defined as cigarette smokers and included in this study. However, when they answered “never” or “used to smoke”, they were defined as noncigarette smokers and excluded.

Cigarette smoking participants were then defined as dual users of e-cigarettes and cigarettes or not based on their responses to the question, “How would you describe your e-cigarette use?”. When they answered “using”, they were defined as dual users. However, when they answered “never” or “used to smoke”, they were defined as cigarette-only smokers.

#### 2.4.2. Cigarette Smoking and E-Cigarette Use Behaviors

Smoking behaviors were evaluated in terms of age at cigarette smoking initiation (9–17/18–24 years), monthly expense for all tobacco products (≤1000/>1000 Thai baht [THB]), daily cigarette smoker (no/yes), past year’s cigarette quit attempt (no/yes), cigarette quit intention (no/yes), and reasons for using e-cigarettes.

The Heaviness of Smoking Index (HSI) score was used to identify the level of nicotine dependence. The overall HSI score ranged between zero and six, with six representing the highest nicotine dependence level (0–2; low/3–4; moderate/5–6; high). Answers to two questions were used to calculate this score, namely time to first cigarette after waking up (≤5 min; 3 scores/6–30 min; 2 scores/31–60 min; 1 score/≥61 min; 0 score) and daily cigarette consumption (1–10 cigarettes; 0 score/11–20 cigarettes; 1 score/21–30 cigarettes; 2 scores/≥31 cigarettes; 3 scores) [27].

#### 2.4.3. Sociodemographic Characteristics

Sociodemographic characteristics were evaluated in terms of sex (female/male), age (years), academic year of study (first/second/third/fourth and higher), monthly income (≤10,000/>10,000 THB), accommodation (stay with parents/on campus housing/off campus housing), living with others (alone/friends/boyfriend/girlfriend/parents/relatives), medical conditions (no/yes), and daily alcohol consumption (no/yes).

### 2.5. Statistical Analysis

Continuous data were described using means and standard deviation (SD) and categorical data were described using frequency and percentage. Differences between two independent groups (dual users and cigarette-only smokers) were compared using independent *t*-test for continuous data and Fisher’s exact test for categorical data. Binary logistic regression analysis was employed to determine factors associated with the dual use of e-cigarettes and cigarettes, and the strength of association was computed using adjusted odds ratio (aOR) and 95% confidence intervals (CI). All exploratory variables, associated with the dual use using univariable logistic regression analysis (*p* < 0.05), were included in a multivariable logistic regression analysis. No multicollinearity was observed among independent variables in the final model [28]. In addition, the goodness of fit of the final model was evaluated using the Hosmer–Lemeshow test. Data with a significance level were defined as two-tailed with a *p* < 0.05. All analyses were computed using STATA Software, Version 14 (StataCorp LP, College Station, TX, USA).

## 3. Results

### 3.1. General Characteristics and Cigarette Smoking Behaviors

Of the 494 participants who were cigarette smokers, 409 participants were dual users (82.8%). About one half of the participants were male (51.0%; *n* = 252) with an average age of 21.40 years (SD 1.20) and smoked less than or equal to 10 cigarettes daily (50.2%; *n* = 248). Most initiated their first cigarette smoking at 17 years or younger (78.3%; *n* = 387) and expressed a moderate level of nicotine dependence evaluated by HSI score (66.2%; *n* = 327). Almost all reported daily cigarette smoking (92.9%; *n* = 459). Interestingly, only a few reported past year’s cigarette quit attempts (8.1%; *n* = 40) and cigarette quit intention (4.4%; *n* = 22) (Table 1 and Table 2).

### 3.2. Factors Associated with the Dual Use of E-cigarettes and Cigarettes

**Figure 1:**
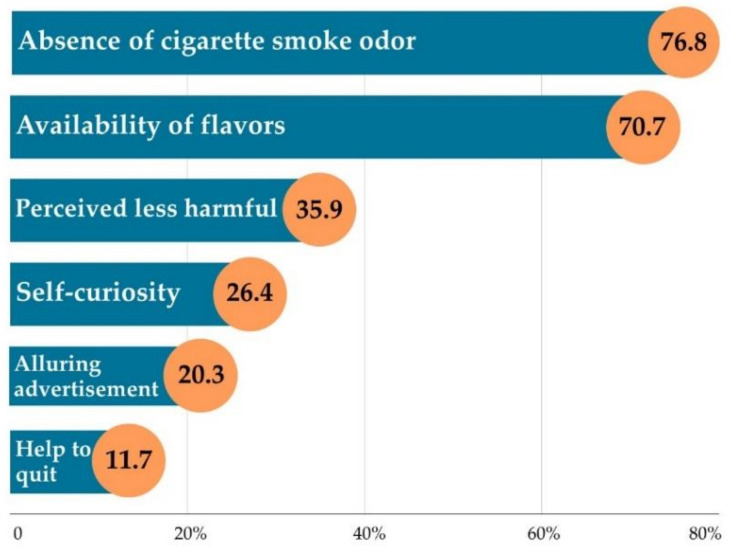
Reasons for using e-cigarette (*n* = 409).

Undergraduate students using e-cigarettes reported two main reasons, including an absence of cigarette smoke odor (76.8%; *n* = 314) and availability of flavors (70.7%; *n* = 289). The perceived less harmful effects than from other forms of tobacco product, especially cigarettes, was third but at a much lower rate (35.9%; *n* = 147), while help to quit cigarette smoking was last (11.7%; *n* = 48) (Figure 1). 

Using univariable logistic regression analysis, factors including initiating cigarette smoking at 18 years or older compared with younger, spend more than 1000 THB for all tobacco products monthly compared with fewer amounts and smoking more than or equal to 11 cigarettes daily compared with fewer amounts were significantly associated with being more likely to be dual users. On the contrary, factors including having a medical condition, consuming alcohol daily, and having past year’s cigarette quit attempts were significantly associated with being less likely to be dual users (Table 3).

After determining multicollinearity and goodness of fit, the final model from multivariable logistic regression analysis revealed three factors significantly associated with the dual use. Undergraduate smokers who initiated smoking at 18 years or older were more likely to be dual users than those who initiated smoking at 17 years old or younger (aOR: 2.79, 95% CI: 1.32–5.89, *p* = 0.007). Moreover, undergraduate smokers who smoked more than or equal to 11 cigarettes daily were more likely to be dual users than those who smoked less than or equal to 10 cigarettes daily (aOR: 2.64, 95% CI: 1.52–4.61, *p* = 0.001). Conversely, undergraduate smokers having past year’s cigarette quit attempt were less likely to be dual users (aOR: 0.26, 95% CI: 0.12–0.56, *p* = 0.001) (Table 3).

## 4. Discussion

### 4.1. Principal Findings

This study constituted one of the few in Thailand focused on the dual use of e-cigarettes and cigarettes among undergraduate students who smoked cigarettes. The finding that 82.8% of the undergraduate smokers were dual users was much higher than that reported in related studies (5.0–19.0%) [7,8,12,22]. This could be explained by different definitions of e-cigarette use. This study defined e-cigarette use by self-report by those responding that they had used e-cigarettes. Related studies have focused on restricting e-cigarette users to those who used them daily or occasionally [7], used them in the last 30 days [8,12], or used at least one e-cigarette cartridge or four drops of e-liquid in the last 12 months [22]. However, regarding less conservative definitions of e-cigarette use, this study could assess a broader population of e-cigarette users.

Noteworthy, in Thailand, e-cigarettes have been outlawed since 2014 [29]. E-cigarettes are an embargoed good; importation of any e-cigarette or any equipment associated with e-cigarettes, such as the liquid used in the device, is prohibited. The retail sale of e-cigarettes is also prohibited. In addition, using e-cigarettes in a smoke-free area is illegal, according to the Tobacco Products Control Act of 2017. However, Thailand encounters the illegal distribution of e-cigarettes, particularly via online platforms and social media, which is a strong driver for the sale and marketing of e-cigarettes [29]. One related study reported that friends and local websites were the main sources of e-cigarettes among university students [30]. As a result, these findings emphasized the importance of increasing and vigorously enforcing an implementation of e-cigarette regulations, particularly in terms of advertising and online distribution.

Undergraduate students, who used e-cigarettes, reported two main reasons: the absence of cigarette smoke odor and the availability of flavors, while a small number of undergraduate students used e-cigarettes as a smoking cessation aid. This finding was consistent with related studies in which undergraduate students did not use e-cigarettes primarily for quitting smoking [7,8]. Other frequently described reasons for e-cigarette use by undergraduate students included self-curiosity [7], enjoyment [8], and using e-cigarettes as a less dangerous substitute to conventional cigarettes [31]. Only a few studies revealed that university students primarily use e-cigarettes to assist smoking cessation [12,31]. This could be ascribed to the fact that e-cigarettes were available in a wide range of flavors [1,2]. These flavorings made e-cigarettes palatable and attractive to undergraduate students. Curiosity was a strong driver of e-cigarette experimentation, and the enjoyment was a dramatic reason for continuing use. Moreover, the absence of cigarette smoke odor when used might make e-cigarettes appear less harmful than conventional cigarettes. Therefore, these findings highlighted the importance for public health and healthcare professionals to provide accurate information to undergraduate students regarding the dangers of e-cigarette use.

The findings indicated three factors significantly associated with the dual use of e-cigarettes and cigarettes, including older age at cigarette smoking initiation, a higher number of cigarettes smoked daily, and having no past year’s cigarette quit attempts. The association between older age at cigarette smoking initiation and the dual use observed in this study contrasted with a related study among young adults in the US [22]. Although the cause of this difference remained uncertain, differences in participant characteristics may have influenced the determination of this association. However, the finding may represent the sequence of dual use. One limitation of this study was that the exact time of initiating cigarettes and e-cigarettes was not collected. Dual users may start with e-cigarette use, then follow by cigarette smoking. E-cigarettes may open up opportunities to use conventional cigarettes and other tobacco products [1,19,30]. Although starting cigarette smoking at a later age has been strongly related to a lower risk of lifelong smoking, nicotine addiction, respiratory and cardiovascular health consequences, and other adverse smoking-related health conditions, every tobacco product including e-cigarettes is not without danger [1,32]. Tobacco control policies should prioritize addressing young populations to avoid initiating all kind of tobacco products.

The association between a higher number of cigarettes smoked daily and the dual use observed in this study was consistent with a related study among young adults in the US [22] and a study among university students in Poland [23]. This finding suggested the necessity to be concerned that dual use could lead undergraduate students to heavier cigarette smoking and higher nicotine addiction because the use of e-cigarettes could also expose them to nicotine. Moreover, nicotine addiction could also lead them to use other nicotine-containing products.

The association between having no past year’s cigarette quit attempts and the dual use observed in this study yielded mixed findings with related studies. This finding was consistent with a study of young adults in the US [22]. On the contrary, a study of university students in France [7] and a study of young adults in China [31] discovered that attempts to quit smoking were more frequently reported among dual users. This inconsistency may be attributed to differences in participant characteristics, adjusted or unadjusted findings, and the number and type of covariates considered in association analysis in the studies. However, this finding strengthens the reasons for using e-cigarettes identified in this study, revealing that undergraduate students did not use e-cigarettes primarily to quit smoking.

### 4.2. Practice Implications of This Study

This study found that undergraduate students used e-cigarettes because of the absence of cigarette smoke odor and availability of flavors. Moreover, they perceived that e-cigarette was less harmful than other forms of tobacco product, especially conventional cigarettes. Therefore, all relevant organizations, particularly universities, should prioritize comprehensive educational programs that convey accurate information regarding the dangers and negative health effects of e-cigarettes. The programs should emphasize that all forms of tobacco products, including e-cigarettes, are not without harm, although they do not have any smoke odor. Moreover, the programs should communicate that e-cigarettes represent an embargoed good in Thailand. The findings also indicated that dual use of e-cigarettes and cigarettes was associated with starting cigarette smoking at ≥18 years old, which was the age that undergraduate students approximately entered universities. This suggested that universities should implement an intensive anti-smoking program, and all university areas should be smoke-free from all tobacco products, including e-cigarettes.

### 4.3. Limitations

Several potential limitations were encountered in this study. First, the self-reported data could represent potential recall bias concerning reporting cigarette smoking and e-cigarette use behaviors. Second, the study defined cigarette smoking and e-cigarette use status by answers from undergraduate students who were using them without biochemical verification. These might have limited the comparability of the findings with other studies that defined them differently. Third, the study did not collect the exact time of initiating cigarettes and e-cigarettes. Therefore, the finding was unable to indicate the probable sequence between cigarette smoking and e-cigarette use in dual users. Finally, the study was restricted to a group of undergraduate students from northern Thailand. As a result, the findings may be limited in their applicability to undergraduate students in other areas of the country.

## 5. Conclusions

This study extended recent findings on factors associated with the dual use of e-cigarettes and cigarettes among undergraduate students who smoked cigarettes. Undergraduate smokers mainly used e-cigarette because of the absence of cigarette smoke odor and the availability of flavors. They did not use e-cigarettes primarily as a cigarette smoking quitting aid. Three factors were associated with the dual use, including older age at cigarette smoking initiation, a higher number of cigarettes smoked daily, and having no past year’s cigarette quit attempts.

## Figures and Tables

**Table 1:** Sociodemographic characteristics of the 494 participants.

Characteristic	All Participants (*n* = 494), *n* (%)	Dual Users of E-Cigarette and Cigarette (*n* = 409), *n* (%)	Cigarette-Only Smokers (*n* = 85), *n* (%)	*p* Value
Sex				
Female	242 (49.0)	201 (49.1)	41 (48.2)	0.906
Male	252 (51.0)	208 (50.9)	44 (51.8)	
Age (years), mean (SD)	21.40 (1.20)	21.42 (1.24)	21.33 (1.03)	0.499
Academic year of study				
First	42 (8.5)	37 (9.0)	5 (5.9)	0.452
Second	69 (14.0)	53 (13.0)	16 (18.8)	
Third	91 (18.4)	77 (18.8)	14 (16.5)	
Fourth and higher	292 (59.1)	242 (59.2)	50 (58.8)	
Monthly income (THB) ^1^				
≤10,000	233 (47.2)	185 (45.2)	48 (56.5)	0.073
>10,000	261 (52.8)	224 (54.8)	37 (43.5)	
Accommodation				
With parents	57 (11.5)	48 (11.7)	9 (10.6)	0.733
On campus housing	17 (3.4)	13 (3.2)	4 (4.7)	
Off campus housing	420 (85.0)	348 (85.1)	72 (84.7)	
Living with others				
Alone	97 (19.6)	82 (20.0)	15 (17.6)	0.705
Friends	195 (39.5)	158 (38.6)	37 (43.5)	
Boyfriend/girlfriend	146 (29.6)	120 (29.3)	26 (30.6)	
Parents/Relatives	56 (11.3)	49 (12.0)	7 (8.2)	
Medical conditions				
No	482 (97.6)	402 (98.3)	80 (94.1)	0.039
Yes	12 (2.4)	7 (1.7)	5 (5.9)	
Daily alcohol consumption				
No	159 (32.2)	140 (34.2)	19 (22.4)	0.041
Yes	335 (67.8)	269 (65.8)	66 (77.6)	

SD: standard deviation; THB: Thai baht; ^1^ 1 USD = 32 THB.

**Table 2:** Smoking behaviors among dual users of e-cigarette and cigarette and cigarette-only smokers.

Smoking Behavior	All Participants (*n* = 494), *n* (%)	Dual Users of E-Cigarette and Cigarette (*n* = 409), *n* (%)	Cigarette-Only Smokers (*n* = 85), *n* (%)	*p* Value
Age at cigarette smoking initiation (years)				
9–17	387 (78.3)	312 (76.3)	75 (88.2)	0.014
18–24	107 (21.7)	97 (23.7)	10 (11.8)	
mean (SD)	15.13 (2.46)	15.31 (2.46)	14.29 (2.33)	<0.001
Monthly expense for all tobacco products (THB) ^1^				
≤1000	162 (32.8)	123 (30.1)	39 (45.9)	0.007
>1000	332 (67.2)	286 (69.9)	46 (54.1)	
Daily cigarette smoker				
No	35 (7.1)	26 (6.4)	9 (10.6)	0.167
Yes	459 (92.9)	383 (93.6)	76 (89.4)	
Daily cigarette consumption				
1–10	248 (50.2)	186 (45.5)	62 (72.9)	<0.001
11–20	206 (41.7)	187 (45.7)	19 (22.4)	
21–30	34 (6.9)	31 (7.6)	3 (3.5)	
≥31	6 (1.2)	5 (1.2)	1 (1.2)	
Time to first cigarette after waking up (minutes)				
≤5	269 (54.4)	218 (53.3)	51 (60.0)	0.767
6–30	146 (29.6)	123 (30.1)	23 (27.1)	
31–60	57 (11.5)	49 (12.0)	8 (9.4)	
≥61	22 (4.4)	19 (4.6)	3 (3.5)	
Heaviness of Smoking Index (HSI) score ^2^				
0–2 (low nicotine dependence)	141 (28.5)	110 (26.9)	31 (36.5)	0.221
3–4 (moderate nicotine dependence)	327 (66.2)	277 (67.7)	50 (58.8)	
5–6 (high nicotine dependence)	26 (5.3)	22 (5.4)	4 (4.7)	
Past year’s cigarette quit attempt				
No	454 (91.9)	384 (93.9)	70 (82.4)	0.001
Yes	40 (8.1)	25 (6.1)	15 (17.6)	
Cigarette quit intention				
No	472 (95.6)	392 (95.8)	80 (94.1)	0.560
Yes	22 (4.4)	17 (4.2)	5 (5.9)	

SD: standard deviation; THB: Thai baht; ^1^ 1 USD = 32 THB; ^2^ The scores ranged between 0 and 6, with 6 representing the highest nicotine dependence level.

**Table 3:** Univariable and multivariable logistic regression analysis of factors associated with the dual use of e-cigarettes and cigarettes among undergraduate smokers.

Factor	Crude OR (95%CI)	*p* Value	Adjusted OR (95%CI)	*p* Value
Medical conditions				
No	1.00		1.00	
Yes	0.28 (0.09–0.90)	0.033	0.41 (0.12–1.42)	0.162
Daily alcohol consumption				
No	1.00		1.00	
Yes	0.55 (0.32–0.96)	0.035	0.84 (0.46–1.52)	0.556
Age at cigarette smoking initiation (years)				
9–17	1.00		1.00	
18–24	2.33 (1.16–4.69)	0.017	2.79 (1.32–5.89)	0.007
Monthly expense for all tobacco products (THB) ^1^				
≤1000	1.00		1.00	
>1000	1.97 (1.22–3.17)	0.005	1.52 (0.91–2.54)	0.109
Daily cigarette consumption				
1–10	1.00		1.00	
≥11	3.23 (1.93–5.42)	<0.001	2.64 (1.52–4.61)	0.001
Past year’s cigarette quit attempt				
No	1.00		1.00	
Yes	0.30 (0.15–0.60)	0.001	0.26 (0.12–0.56)	0.001

OR: odds ratio; CI: confidence intervals; THB: Thai baht; ^1^ 1 USD = 32 THB.

## Data Availability

The data presented in this study are available from the corresponding author on reasonable request.

## Supplementary Materials

The following are available online at https://www.mdpi.com/article/10.3390/children8121197/s1, File S1: The study questionnaire.
