# Supplementary material for: Factors Associated with the Dual Use of Electronic Cigarettes and Cigarettes among Thai Undergraduate Students Who Smoked Cigarettes
Source: Children (Basel). 2021 Dec 17;8(12):1197. doi: 10.3390/children8121197 (PMC8700373; doi:10.3390/children8121197)
Supplement: Supplementary file 1 [file children-08-01197-s001.zip › children-1490554-supplementary.pdf]

The study questionnaire

Online informed consent form

You are invited to participate in an online questionnaire of a study of electronic cigarette (e-cigarette) use among undergraduate students who smoked cigarettes in Chiang Mai Province, Thailand. This study is being conducted by Ms. Phantara Chulasai, a student in the Ph.D.'s Degree Program in Pharmacy at the Faculty of Pharmacy, Chiang Mai University.

This study aimed to assess cigarette smoking, e-cigarette use, and factors associated with the dual use of e-cigarette and cigarette among undergraduate students. To participate in this study, you must be an undergraduate student from universities in Chiang Mai Province, Thailand, 18 years or older, smoking cigarettes, able to complete the online questionnaire on the Google Forms platform, and able to communicate in Thai.

During the study, you will complete an online questionnaire on your demographic information and smoking-related behaviors, which should take about 15 minutes to complete. This study provides no compensation, and you will receive no direct benefits from participating in this study. However, your responses may help the researcher learn more about undergraduate students' cigarette smoking and cigarette smoking behaviors. The risks you take by participating in this study are modest and not greater than those you face in everyday life. You may be offended by some of the questions in the questionnaire. Your participation in this study is entirely voluntary, and you may withdraw at any time without penalty.

This anonymous online questionnaire is being conducted using the Google Forms platform. No identifying information about you is being collected. All information collected will be kept confidential on a password-protected computer and used only for this research. There will be no connection to you specifically in the results or future publication of the results.

If you have any questions or want additional information about this study, please contact Ms. Phantara Chulasai at panthara.chula@gmail.com or 085-029-5363. This questionnaire was reviewed and approved by the Human Ethics Committee of the Faculty of Pharmacy, Chiang Mai University (ethics approval number 023/2018), issued 11 July 2018.

Please select your choice below. Clicking on the "Agree" button indicating that you are 18 years of age or older, have read and understood the information on this form, and voluntarily agree to participate. This link will direct you to the online questionnaire. Clicking on the "Disagree" entry will close this page and you will exit the study.

☐	☐
Agree	Disagree
I agree to participate.	I do not wish to participate at this time.

Your responses are very important for this study. Please read each question carefully and select only one answer for each question that best pertains to you.

Part 1: Cigarette smoking status

1. **How would you describe your cigarette smoking habits?**
   - ○ Smoking *(continue to the questionnaire Part 2)*
   - ○ Never *(end of the questionnaire)*
   - ○ Used to smoke *(end of the questionnaire)*

Part 2: Sociodemographic characteristics

2. What is your sex?
   - ○ Female
   - ○ Male
3. How old are you now?

   _______ years
4. What is your academic year of study?
   - ○ 1
   - ○ 2
   - ○ 3
   - ○ 4 and higher
5. How much money do you receive from your parents monthly?
   - ○ ≤10,000 Thai Baht
   - ○ >10,000 Thai Baht
6. Where do you reside during the university semesters?
   - ○ With parents
   - ○ On campus housing
   - ○ Off campus housing

7. With whom do you reside?

- ○ Alone
- ○ Friends
- ○ Boyfriend/girlfriend
- ○ Parents/ Relatives

8. Do you have any medical conditions?

- ○ No
- ○ Yes

9. Do you consume alcohol every day?

- ○ No
- ○ Yes

Part 3: Cigarette smoking behaviors

10. How old were you when you first started smoking cigarettes?

    ________ years

11. How much money do you spend on all tobacco products?

- ○ ≤1,000 Thai Baht
- ○ >1,000 Thai Baht

12. Do you smoke cigarettes every day during the past month?

- ○ No
- ○ Yes

13. How many cigarettes do you smoke per day?

- ○ 1–10
- ○ 11–20
- ○ 21–30
- ○ ≥31

14. How soon after waking up do you smoke your first cigarette?

- ○ ≤5 minute
- ○ 6–30 minute
- ○ 31–60 minute
- ○ ≥61 minute

15. Have you attempted to quit cigarette smoking during the past year?
  - ○ No
  - ○ Yes
16. Do you intend to quit cigarette smoking?
  - ○ No
  - ○ Yes

Part 4: E-cigarette use

17. How would you describe your e-cigarette use?
  - ○ Using *(continue to the question number 18)*
  - ○ Never use *(end of the questionnaire)*
  - ○ Used to use *(end of the questionnaire)*
18. What is your reason for using e-cigarettes?
  - ○ Absence of cigarette smoke odor
  - ○ Availability of flavors
  - ○ Perceived less harmful than other forms of tobacco product, especially cigarettes
  - ○ Self-curiosity
  - ○ Alluring advertisements
  - ○ Help to quit cigarette smoking

End of the questionnaire. Thank you very much for participating in this study.
